# Supplementary material for: COVID-19 vaccine brand hesitancy and other challenges to vaccination in the Philippines
Source: PLOS Glob Public Health. 2022 Jan 13;2(1):e0000165. doi: 10.1371/journal.pgph.0000165 (PMC10021706; doi:10.1371/journal.pgph.0000165)
Supplement: Attachment — Submitted filename: Response to Reviewers.docx [file pgph.0000165.s001.docx]

08 December 2021

**RE: PLOS Global Health – Revisions to Manuscript ID PGPH-D-21-00884**

Dear Dr. Dione Benjumea-Bedoya:

We would like to thank the editorial team of PLOS Global Health and the peer reviewers for considering our manuscript, entitled *“***COVID-19 vaccine brand hesitancy and other challenges to vaccination in the Philippines**” (**Manuscript ID PGPH-D-21-00884**). We believe that the feedback provided, all points of which have been accepted and implemented in full (see point-by-point table of responses below) or responded to, has allowed us to greatly improve the manuscript.

Please do not hesitate to contact me if you have any questions, and we thank you for your continued consideration of our revised manuscript.

Yours truly,

Arianna Maever L Amit, on behalf of my co-authors

**AUTHOR RESPONSES TO COMMENTS**

**Editor**

| **Comment** | **Author response** |
| --- | --- |
| The authors presented a complete and very good quality work, which contributes to broadening the knowledge about COVID-19 vaccine hesitancy. The reviewers agree on the relevance of this manuscript as well as its good quality, and made some recommendations that are presented below and in the attached file, so that the manuscript becomes suitable to be considered for publishing. | Thank you very much for this comment. We have revised the manuscript according to the recommendations. We have also noted that no copyediting will be made, and have made minor edits on the text and headings to improve on flow and clarity. |

**Reviewer 1**

| **Comment** | **Author response** |
| --- | --- |
| Reviewer #1: The article presented is of excellent quality, urgent and necessary to understand the complexity in the face of hesitations to vaccinate for Covid-19.  The authors designed a coherent qualitative study that, through semi-structured interviews, content analysis and grounded theory, managed to reach pertinent and coherent conclusions regarding the individual, interpersonal and structural reasons for not accepting vaccination in the Philippines.  The authors are very organized in their analysis strategy and presentation of results that allow coherently linking the categories with the testimonies that support them, organized in the three main dimensions of reasons: individual, interpersonal and structural.  Promoting this multilevel understanding should guide health authorities to guide multilevel actions in the face of the complexity of hesitations regarding vaccination for covid-19, not only in the Philippines, but also in similar social and cultural contexts. | Thank you very much for this comment. |

**Reviewer 2**

| **Comment** | **Author response** |
| --- | --- |
| Reviewer #2: It is a very good job, the situation described in the Philippines is similar to other places in the world.  I consider that you should make clear how you avoided the loss of information when translating from Filipino and Cebuano to English.  You should revise reference number 2, it is not in accordance with the standard. | Thank you very much for this comment. We have revised the paper to clarify how we avoided loss of information during the translation process and to correct citation of reference 2. |
| Data analysis, P. 10, Line 218: How you avoided the loss of information in translation? | The research team are native and/or fluent speakers of the three languages, and checked for linguistic and conceptual equivalence in the translated documents. |
| Data analysis, P. 11, Line 237: Have you used any software for the analysis? | We used Microsoft Excel and have mentioned and cited this in the text. |
| Discussion, P. 32, Lines 499-500: Could you explore further the influence of these actors on vaccination? | The influence of these actors are discussed in detail in the succeeding paragraphs. Briefly, social networks may either positively or negatively impact vaccination uptake, depending on their views on vaccines. Political issues and perceptions about the government’s response contribute to vaccine brand hesitancy, resulting to vaccination delays and refusals individual. We have also provided specific recommendations relating to these actors in the Conclusion. |
| Conclusions, P. 36, Lines 577-579: According to the results, what kind of government interventions should they include? Use of media, educational campaigns, scientific literature? | We asked participants what their recommendations were for the vaccination program in the country. While this paper focuses on the challenges and not the potential opportunities, we included some specific recommendations in the Conclusion as suggested by the reviewer. |
| References, P. 38, Reference 2: Review reference | We have corrected the citation of reference 2. |

**Journal requirements**

| **Comment** | **Author response** |
| --- | --- |
| 1. Please review your reference list to ensure that it is complete and correct. If you have cited papers that have been retracted, please include the rationale for doing so in the manuscript text, or remove these references and replace them with relevant current references. Any changes to the reference list should be mentioned in the rebuttal letter that accompanies your revised manuscript. If you need to cite a retracted article, indicate the article’s retracted status in the References list and also include a citation and full reference for the retraction notice. | We have checked that our reference list is complete and correct. |
| 2. Please update the completed 'Competing Interests' statement, including any COIs declared by your co-authors. If you have no competing interests to declare, please state "The authors have declared that no competing interests exist". Otherwise please declare all competing interests beginning with the statement "I have read the journal's policy and the authors of this manuscript have the following competing interests: | We have updated the competing interests statement. |
| 3. We see that your study includes live participants, but you have not included an Ethics Statement. Please update your manuscript file to include an Ethics Statement subsection to your Materials and Methods section. It should include:  iii) (for human participants or donors) - A statement that formal consent was obtained (must state whether verbal/written) OR the reason consent was not obtained (e.g. anonymity) | We have included information about the consent obtained in our manuscript. |
| 4. Please provide separate figure files in .tif or .eps format only and remove any figures embedded in your manuscript file. Please ensure that all files are under our size limit of 20MB.  For more information about how to convert your figure files please see our guidelines: Once you've converted your files to .tif or .eps, please also make sure that your figures meet our format requirements | We have converted our figure to .tif. |
| 5. Please amend your detailed Financial Disclosure statement. This is published with the article, therefore should be completed in full sentences and contain the exact wording you wish to be published.  ii). State the initials, alongside each funding source, of each author to receive each grant.  iii). State what role the funders took in the study. If the funders had no role in your study, please state: “The funders had no role in study design, data collection and analysis, decision to publish, or preparation of the manuscript.”  iv). If any authors received a salary from any of your funders, please state which authors and which funders. | We have revised our financial disclosure statement. |
